# Pathological Aging of Patients With Amyotrophic Lateral Sclerosis: A Preliminary Longitudinal Study

**DOI:** 10.1002/brb3.70484

**Published:** 2025-05-07

**Authors:** Sana Mohammadi, Sadegh Ghaderi, Farzad Fatehi, Sanjay Kalra, Seyed Amir Hossein Batouli

**Affiliations:** ^1^ Neuromuscular Research Center, Department of Neurology Shariati Hospital, Tehran University of Medical Sciences Tehran Iran; ^2^ Department of Neuroscience and Addiction Studies, School of Advanced Technologies in Medicine Tehran University of Medical Sciences Tehran Iran; ^3^ Neuroscience and Mental Health Institute University of Alberta Edmonton Alberta Canada; ^4^ Division of Neurology, Department of Medicine University of Alberta Edmonton Alberta Canada

**Keywords:** aging, ALS, deep learning, structural MRI

## Abstract

**Objective:**

This longitudinal study investigated pathological brain aging in amyotrophic lateral sclerosis (ALS) by evaluating disparities between chronological age and deep learning‐derived brain structure age (BSA) and exploring associations with cognitive and functional decline.

**Methods:**

Ten limb‐onset ALS patients (seven males) and 10 demographically matched healthy controls (HCs) underwent structural magnetic resonance imaging (sMRI) and cognitive assessments at baseline and follow‐up. The BSA was estimated using the validated volBrain platform. Cognitive domains (language, verbal fluency, executive function, memory, and visuospatial skills) and global cognition (Persian adaptive Edinburgh Cognitive and Behavioral ALS Screen [ECAS] total score) were assessed along with functional status (ALSFRS‐R).

**Results:**

ALS patients exhibited significant BSA‐chronological age disparities at baseline (Δ = +7.31 years, *p* = 0.009) and follow‐up (Δ = +8.39 years, *p* = 0.003), with accelerated BSA progression over time (*p* = 0.004). The HCs showed no such disparities (*p* = 0.931). Longitudinal BSA increases were correlated with executive function decline (*r* = −0.651, *p* = 0.042). Higher education predicted preserved language (*r* = 0.831, *p* = 0.003) and verbal fluency (*r* = 0.738, *p* = 0.015). ALSFRS‐R decline paralleled visuospatial (*r* = 0.642, *p* = 0.045) and global cognitive deterioration (*r* = 0.667, *p* = 0.035).

**Conclusions:**

ALS is characterized by accelerated structural brain aging that progresses independently of chronological age and is correlated with executive dysfunction. Education may mitigate cognitive decline, while motor functional deterioration aligns with visuospatial and global cognitive impairments. BSA has emerged as a potential biomarker for tracking pathological aging trajectories in ALS, warranting validation using larger cohorts.

## Graphical abstract

This study utilizes a deep learning approach called Brain Structure Ages (BSA) to demonstrate accelerated and progressive biological brain aging in patients with amyotrophic lateral sclerosis (ALS), highlighting its potential as a valuable tool for understanding disease progression and informing future therapeutic strategies.

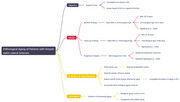

## 1 Introduction

Amyotrophic lateral sclerosis (ALS) is a progressive neurodegenerative disease that primarily affects the upper and lower motor neurons and has a complex impact on motor and extra‐motor structures (Christidi et al., 2018; Hardiman et al., 2017). ALS leads to debilitating muscle weakness, wasting, and other features of motor neuron dysfunction (Fatehi et al., 2018). It often progresses rapidly, with an average survival of 2–5 years after onset (Masrori and Van Damme, 2020). Treatment options are limited, and no cure is currently available (Tzeplaeff et al., 2023). Although ALS can affect individuals of various ages, the risk increases significantly with advancing age (Ingre et al., 2015). Notably, both sporadic and genetic forms of ALS exhibit pathophysiological characteristics that overlap with the hallmarks of aging, including genomic instability, DNA damage, mitochondrial dysfunction, inflammation, proteostasis, and cellular senescence (Dashtmian et al., 2024; Jagaraj et al., 2024). Although supportive care remains the mainstay of ALS management, recent years have witnessed the emergence of disease‐modifying therapies. Riluzole (Bensimon et al., 1994), a glutamate release inhibitor, and edaravone (Writing Group, Edaravone (MCI‐186) ALS 19 Study Group 2017), a free radical scavenger, have demonstrated modest survival benefits in sporadic ALS. In addition, tofersen, a novel therapy targeting superoxide dismutase 1 (SOD1) protein levels, has been approved for familial ALS (Miller et al., 2022). Although promising, these advancements highlight the ongoing need for more effective treatments to address the complex pathophysiology of ALS related to aging and other ALS hallmarks.

Numerous biological aging processes are differentially regulated in neurodegenerative diseases, including mitochondrial dysfunction, aberrant autophagy, epigenetic alterations, and inflammation (Gonzales et al., 2022). Aging is the most significant risk factor for ALS, with an average age of 55 years at ALS diagnosis (Jagaraj et al., 2024). Age‐related dysfunction at the neuromuscular junction may represent a key pathological mechanism in ALS. The overlap between aging and ALS‐associated hallmarks suggests that cell type‐specific aging may be a critical contributor to this multifactorial and complex disease (Jagaraj et al., 2024).

**FIGURE 1:**
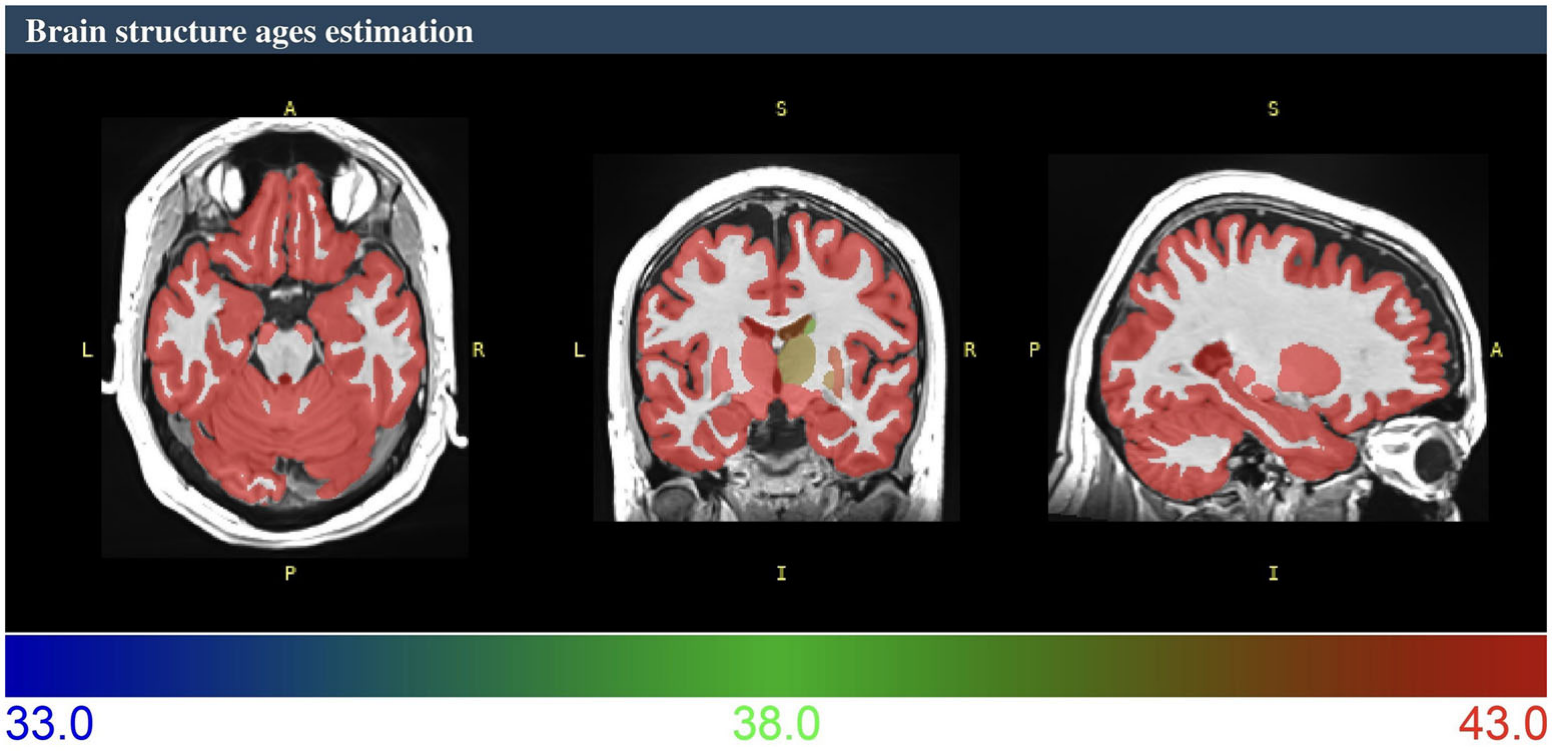
A sample analysis of structural T1‐weighted MRI for brain structure age (BSA) estimation using the volBrain platform (range represents years; the chronological age of this patient was 38 years at baseline).

Aging is associated with several structural and functional changes in the brain, such as gray and white matter atrophy, changes in cerebrospinal fluid and ventricular volumes, and alterations in white matter microstructural integrity (Coelho and Sousa, 2022). These changes are linked to cognitive decline and increased risk of neurodegenerative disorders. In ALS, specific brain regions, including the motor cortex, basal ganglia, and cerebellum, are affected by neurodegeneration (Menke et al., 2014; Ghaderi et al., 2024). Understanding how these brain structural changes are related to aging and ALS progression is critical for developing accurate diagnostic tools and effective therapies. Recently, a novel deep learning‐based approach, brain structure ages (BSA), was introduced to estimate the age of individual brain structures (Figure 1) (Nguyen et al., 2024). BSA, a measure of how an individual's brain structure compares with the expected norm for chronological age, offers valuable insights into the progression of neurological, neuropsychiatric, and neurodegenerative diseases. BSA was determined by analyzing structural magnetic resonance imaging (sMRI) data, initially creating a voxel‐wise brain‐age map. This detailed map was then used to calculate the average predicted age of various brain structures to produce the BSA. Therefore, because aging induces heterogeneous changes in the brain anatomy, deviations from the typical aging trajectory may indicate underlying neurological conditions, making BSA highly valuable (Nguyen et al., 2024).

Consequently, our study aimed to (1) investigate discrepancies between estimated BSA and chronological age in patients with ALS, (2) determine whether BSA accelerates over time compared to healthy aging, and (3) explore the associations between accelerated brain aging and longitudinal cognitive and functional decline.

## 2 Methods

### 2.1 Ethics Approval and Participants

The Ethics Committee of Tehran University of Medical Sciences approved this study (Ethical Code: *IR.TUMS.MEDICINE.REC.1400.1173*). Written informed consent was obtained from all participants. A total of 10 patients with ALS were recruited and followed up, undergoing clinical examinations to confirm the absence of a history of neurological, psychiatric, or cognitive issues. Standard assessments included clinical interviews, neurological examinations, and sMRI. Participants with a history of sensory complaints, other nervous system disorders, or psychiatric or brain trauma were excluded. Diagnoses were confirmed through a multidisciplinary consensus, adhering to the Awaji criteria (Costa et al., 2012), with patients maintaining their routine medications.

Cognitive assessments were conducted using a standardized battery, as detailed in our prior work (Ghaderi et al., 2024), to evaluate domain‐specific functions, including language, verbal fluency, executive functioning, memory, and visuospatial abilities. The Persian‐adapted Edinburgh Cognitive and Behavioral ALS Screen (ECAS) was used to capture the cognitive profiles.

### 2.2 MRI

#### 2.2.1 Acquisition

MRI images were obtained using a Siemens Prisma Scanner (2016), equipped with a 64‐channel GRAPPA head coil. High‐resolution structural data were captured using a 3D T1‐weighted MPRAGE sequence with 176 axial slices. Imaging parameters included repetition time, 1840 ms; echo time, 2.43 ms; inversion time, 900 ms; slice thickness, 1 mm; flip angle, 8°; and field of view, 255 mm^2^ × 255 mm^2^.

#### 2.2.2 Image Analysis and Brain Structure Ages (BSA) Measurements

Initially, T1‐weighted image data were processed using the mri_convert command (https://surfer.nmr.mgh.harvard.edu/fswiki/mri_convert) to adjust voxel size, ensuring isotropic voxels (1 mm × 1 mm × 1 mm (Fatehi et al., 2018)), as required for BSA (Nguyen et al., 2024; de Senneville et al., 2020) analysis (https://volbrain.net/services/BrainStructureAges). Subsequently, using the free online platform volBrain (Manjón and Coupé, 2016) (https://volbrain.net/), we implemented the BSA measurement, a pipeline specifically designed to automatically estimate a biological subject's age through T1‐weighted brain MRI scans. The BSA methodology provides age estimation for each brain structure by relying on advanced deep‐learning models. These age groups contributed to determining the overall biological age of the subject. The BSA models were trained using control subjects ranging in age from 0 to 100 years old. In the final step, all brain structures were segmented using AssemblyNet (Coupé et al., 2020). All the processed data and analyses are available upon request from the corresponding authors.

### 2.3 Statistical Analyses

Variable normality was assessed using the Shapiro–Wilk test. To assess differences within the groups, we used a paired *t*‐test for parametric variables to compare chronological age with BSA estimation at baseline, follow‐up, and healthy controls (HCs) and BSA estimation at baseline with BSA estimation at follow‐up. Independent samples *t*‐tests were used for comparisons between patients with ALS and HCs. The magnitude of the difference was determined using the standardized mean difference (SMD) effect size, with common cut‐offs of 0.2 for small, 0.5 for medium, and 0.8 for large effects (Hedges and Olkin, 1985). Correlational analyses were used to assess the associations between biological age estimation and cognitive and functional trajectories (e.g., ALSFRS‐R, cognitive scores). All data are reported as means ± standard deviation (SD). The significance level was set at *p* < 0.05. All statistical analyses were performed using the SPSS V.27.0 (IBM SPSS Statistics) software.

## 3 Results

### 3.1 Overview of Subject Characteristics

The study included 10 limb‐onset ALS patients (seven males) at both baseline and follow‐up, with sex, age, years of education, and body mass index (BMI) matched to HCs (10 participants, 7 males). The cognitive scores across all domains declined numerically from baseline to follow‐up, although these changes were not statistically significant (all *p* > 0.05) (Table 1). Chronological age and BSA estimates at baseline and follow‐up for each patient are presented in Table 2.

**TABLE 1:** Characteristics of the patients with amyotrophic lateral sclerosis (ALS) (baseline and follow‐up) and healthy controls (HCs).

	Baseline	Follow‐up	Healthy control	*p* value^a^
Patients with ALS (limb‐onset)/male	10/7	10/7	10/7	1.00
Chronological age, years	52.41 (13.93)	52.86 (13.95)	45.70 (7.51)	0.353
Years of education	14.40 (3.95)	14.40 (3.95)	18.50 (5.28)	0.075
BMI, kg/m^2^	30.28 (4.61)	29.12 (6.16)	25.60 (3.81)	0.108
Disease duration, months	11.70 (8.80)	15.80 (8.69)	NA	0.309
ALSFRS‐R	41.90 (2.42)	37.40 (2.67)	NA	0.001
Total language score (0–28)	19.80 (6.77)	17.30 (8.13)	NA	0.464
Total verbal fluency score (0–24)	12.40 (8.53)	9.90 (6.23)	NA	0.464
Total executive score (0–48)	24.30 (13.91)	20.90 (12.28)	NA	0.569
Total memory score (0–24)	11.80 (8.05)	10.70 (7.79)	NA	0.760
Total visuospatial score (0–12)	9.50 (3.21)	7.80 (3.68)	NA	0.285
ECAS score (0–136)	77.80 (36.90)	66.60 (35.02)	NA	0.495

^Note:^ Data presented as mean (SD) for continuous variables or *n* counts (%) for categorical variables.

Abbreviations: ALS, amyotrophic lateral sclerosis; ALSFRS‐R, revised ALS functional rating scale; BMI, body mass index; Not applicable NA.

^a^ Statistical significance was set at *p* < 0.05.

**TABLE 2:** Chronological age and brain structure age (BSA) estimates at baseline and follow‐up for each patient with amyotrophic lateral sclerosis (ALS).

	Baseline		Follow‐up	
Sex	Chronological age	Biological age	Chronological age	Biological age
M	59.52	61.59	59.92	63.82
F	28.42	43.71	28.85	46.94
M	42.71	55.98	43.17	55.19
M	38.02	40.79	38.57	42.65
M	53.11	50.16	53.53	51.98
M	67.52	80.33	67.88	80.17
M	71.34	73.09	71.73	74.63
F	64.04	77.61	64.86	78.40
F	56.12	69.87	56.49	72.44
M	43.28	44.09	43.56	46.26

### 3.2 Groups Comparison

We found a significant disparity between chronological age and BSA estimation in ALS patients at both baseline and follow‐up (Table 3). At baseline, patients with ALS had a mean chronological age of 52.41 years (SD = 13.93), while their estimated BSA was significantly higher (mean = 59.72 years, SD = 14.89; *p* = 0.009), with a large SMD (SMD = 1.01). This gap persisted and widened at follow‐up, with chronological age increasing marginally to 52.86 years (SD = 13.95) and BSA rising to 61.25 years (SD = 14.38; *p* = 0.003 vs. chronological age, SMD = 1.20). Longitudinal comparisons revealed a significant acceleration in BSA between the baseline and follow‐up (*p* = 0.004, SMD = 1.18). In contrast, HCs showed no significant difference in chronological age (mean = 45.70 years, SD = 7.51) and BSA (mean = 45.55 years, SD = 11.87; *p* = 0.931, SMD = −0.028). ALS patients exhibited markedly elevated BSA compared to HCs at both baseline (*p* = 0.030, SMD = 1.01) and follow‐up (*p* = 0.016, SMD = 1.14), with large effect sizes highlighting divergence in brain aging trajectories.

**TABLE 3:** Comparison of chronological age and brain structure age (BSA) estimation at baseline and follow‐up in patients with amyotrophic lateral sclerosis (ALS) and healthy controls (HCs).

			Biological age estimation vs. chronological age^a^			Biological age estimation comparisons			
	Chronological age	Biological age estimation	*p* value^c^	*t*	SMD^d^	Comparisons	*p* value	*t*	SMD
Baseline	52.41 (13.93)^b^	59.72 (14.89)	0.009	3.32	1.01	(Baseline vs. follow‐up)^a^	0.004	−3.89	−1.18
Follow‐up	52.86 (13.95)	61.25 (14.38)	0.003	3.95	1.20				
Healthy control	45.70 (7.51)	45.55 (11.87)	0.931	−0.089	−0.028	(Baseline vs. HC)^e^	0.030	2.35	1.01
						(Follow‐up vs. HC)^e^	0.016	2.66	1.14

Abbreviations: ALS, amyotrophic lateral sclerosis; BSA, brain structure age; HCs, healthy controls; SMD, standardized mean difference.

^a^ Paired comparisons were tested using the paired samples *t*‐test for parametric variables.

^b^ Data presented as mean (SD).

^c^ Statistical significance was set at *p* < 0.05.

^d^ The effect size, Hedges' correction, is calculated using the sample standard deviation of the mean difference (cut off; trivial: −0.19 to 0.19; small: 0.2 to 0.49 and −0.49 to −0.2; medium: 0.5 to 0.79 and −0.79 to −0.5; large: ≥ 0.8 and ≤ −0.8).

^e^ Independent comparisons were tested using the independent samples test for parametric variables.

### 3.3 Cognitive and Functional Associations

At baseline, biological age was not significantly correlated with years of education, BMI, disease duration, ALSFRS‐R scores, or cognitive domains (all *p* > 0.05). However, longitudinal follow‐up identified a significant negative correlation between biological age and the total executive score (*r* = −0.651, *p* = 0.042, 95% CI: −0.908 to −0.035). Education years showed robust positive associations with cognitive performance at both time points, particularly with the total language score (baseline: *r* = 0.831, *p* = 0.003; follow‐up: *r* = 0.661, *p* = 0.038) and total verbal fluency score (baseline: *r* = 0.727, *p* = 0.017; follow‐up: *r* = 0.738, *p* = 0.015). ALSFRS‐R scores correlated with the total memory score at baseline (*r* = 0.796, *p* = 0.006), total visuospatial score (*r* = 0.642, *p* = 0.045), and ECAS score (*r* = 0.667, *p* = 0.035) at follow‐up.

## 4 Discussion

BSA estimation offers a more individualized assessment, potentially enabling the identification of patients at higher risk of rapid disease progression. Our results demonstrated a significant discrepancy between chronological age and BSA estimates in patients with ALS, both at baseline and follow‐up, while there were no differences in HCs. This disparity, characterized by a substantial elevation in BSA relative to chronological age, underscores the accelerated pace of biological aging in these individuals. The consistent increase in BSA estimates over time further supports the notion of progressive biological deterioration in ALS (Dashtmian et al., 2024). Such disparities between chronological and biological age have been reported in other neurodegenerative diseases, where systemic and cellular stressors contribute to an accelerated aging phenotype (Gonzales et al., 2022; Guo et al., 2022; López‐Otín et al., 2023). In ALS, this may reflect the cumulative effects of neuroinflammation, oxidative stress, and mitochondrial dysfunction, all of which are hallmark features of the disease (Dashtmian et al., 2024; Obrador et al., 2020; Zhang et al., 2023; Xiong et al., 2022).

The observation that BSA estimates are significantly higher than chronological age in patients with ALS aligns with the well‐established link between age and ALS. Aging is considered the most prominent risk factor for ALS (Jagaraj et al., 2024); risk before the age of 40 is very low, peaks after midlife (Mehta et al., 2022), and appears to decline in very old adults with a peak incidence between 70 and 79 years of age (Ingre et al., 2015; Alonso et al., 2009; Huisman et al., 2011). The incidence of ALS contrasts with that of other age‐related neurodegenerative diseases (Dashtmian et al., 2024) such as Alzheimer's disease (Mukadam et al., 2024; Kukull et al., 2002) and Parkinson's disease (Van Den Eeden et al., 2003; Willis et al., 2022; Wright Willis et al., 2010), suggesting a unique interplay between aging and ALS pathophysiology (Dashtmian et al., 2024). Furthermore, our findings suggest that ALS does not merely correlate with aging but may actively accelerate the aging process (Jagaraj et al., 2024). Accelerated aging can exacerbate neurodegenerative mechanisms and create a self‐reinforcing cycle that drives disease progression.

The significant progression of BSA between baseline and follow‐up further strengthens the argument for accelerated aging in ALS, and introduces pathological brain aging. This acceleration in biological aging may be attributed to various factors, including the accumulation of cellular damage, such as DNA damage, mitochondrial dysfunction, neuroinflammation, oxidative stress, and cellular senescence (Hou et al., 2019; Mitra et al., 2019; Wang et al., 2016; Lopez‐Gonzalez et al., 2016). In ALS, these processes may be exacerbated by the degeneration of motor neurons and subsequent loss of muscle mass and function (Dashtmian et al., 2024; Campbell et al., 1973). In addition, chronic inflammation, a hallmark of ALS, can accelerate cellular senescence and contribute to systemic aging (Baechle et al., 2023). Thus, the hallmarks of aging, including these factors, are increasingly recognized as potential drivers of both aging and age‐related diseases such as ALS (Guo et al., 2022; Baechle et al., 2023; Shafiq et al., 2021).

The absence of baseline correlations between biological age and cognitive scores may reflect early stage compensatory mechanisms or insufficient disease progression to unmask these relationships, which become apparent longitudinally (Jellinger, 2023). The significant negative correlation between biological age and executive function at follow‐up highlights the time‐dependent relationship between accelerated brain aging and executive dysfunction. This aligns with prior studies implicating frontal–striatal network degeneration in ALS‐related executive impairment (Štukovnik et al., 2010), suggesting that BSA may serve as a biomarker for tracking domain‐specific cognitive decline. The robust association between education and preserved language/verbal fluency performance supports the cognitive reserve hypothesis, wherein higher educational attainment may mitigate the clinical expression of neurodegeneration in ALS (Temp et al., 2022). Notably, the longitudinal association between ALSFRS‐R decline and worsening visuospatial/ECAS scores suggests a coupled progression of motor and cognitive dysfunction, consistent with the concept of ALS as a multisystem disorder (Chiò et al., 2019; Ghaderi et al., 2024).

## 5 Limitations and Directions for Future Research

Although this study focused on BSA as an indicator of accelerated aging in ALS, sources acknowledge the significant role of genetics in this complex disease. Approximately 10% of ALS cases are familial ALS, linked to mutations in various genes, including SOD1, chromosome 9 open reading frame 72 (c9orf72), transactive response DNA‐binding protein 43 kDa (TAR DNA‐binding protein 43 or TDP‐43), and fused in sarcoma/translated in liposarcoma (FUS/TLS) (Shafiq et al., 2021; Volk et al., 2018; Wang et al., 2023). These genes, particularly SOD1, are implicated in critical cellular processes that, when disrupted, contribute to the hallmarks of aging (Guo et al., 2024). For example, SOD1, which is responsible for detoxifying superoxide radicals, plays a role in maintaining genomic stability, a key hallmark of aging (Eleutherio et al., 2021). Mutations in SOD1, commonly found in familial ALS, can lead to toxic gain‐of‐function, causing the accumulation of misfolded SOD1 proteins and disrupting cellular homeostasis (Brasil et al., 2018; Peggion et al., 2022). This disruption potentially accelerates aging by exacerbating cellular stress, impairing proteostasis, and promoting mitochondrial dysfunction (Shafiq et al., 2021). Studies on SOD1 transgenic mice further illustrated the genetic influence on age‐related disease manifestation, with disease onset correlating with the level of mutant SOD1 expression (De Giorgio et al., 2019). While this study did not directly investigate the genetic makeup of the participants, the observed accelerated aging, reflected in the significantly elevated BSA in a relatively young cohort (mean age 52.41 years), points to the potential role of genetic predispositions in driving premature aging in ALS (Shafiq et al., 2021). Further research is needed to explore the complex interplay between specific genetic variants and accelerated aging in ALS, potentially shedding light on the disease subtypes, progression, and personalized treatment strategies.

Although this study offers preliminary evidence supporting the concept of pathological aging in ALS, further research is required to fully elucidate the underlying mechanisms and clinical implications. Future research should investigate the specific brain regions most affected by accelerated aging in ALS using brain age gap estimation (BSAGE) (Nguyen et al., 2024). Furthermore, the limitations of this study, including its small sample size and genetic data, should be addressed in future studies. Expanding the study population and including individuals with various ALS subtypes, such as bulbar‐onset ALS, would enhance the generalizability of our findings. In addition, incorporating other measures of biological aging, such as telomere length and epigenetic clock, could provide a more comprehensive understanding of the aging process (Zhang et al., 2023; Horvath and Ritz, 2015; Salvioli et al., 2023; Rodríguez‐Fernández et al., 2022). The follow‐up period was relatively short, which may have limited the ability to capture long‐term trends in biological aging. Extending the follow‐up period would offer a more thorough understanding of the temporal dynamics of BSA progression in ALS patients.

## 6 Conclusions

Our study revealed accelerated biological aging and confirmed temporal pathological aging in patients with ALS, as evidenced by the significant disparities between BSA estimation and chronological age. The progression of BSA over time underscores the dynamic and compounding nature of aging within the context of ALS pathophysiology, suggesting that targeting the aging process itself could be a promising avenue for future therapeutic interventions aimed at slowing down or reversing the aging process in patients with ALS. These primary neuroimaging findings provide a foundation for future research aimed at uncovering the mechanisms driving accelerated aging in ALS and identifying interventions to mitigate its effects.

## Author Contributions

**Sana Mohammadi**: conceptualization, investigation, writing – original draft, visualization, validation, methodology, software, formal analysis, data curation, resources. **Sadegh Ghaderi**: conceptualization, investigation, methodology, validation, visualization, software, formal analysis, project administration, data curation, supervision, resources, writing – original draft, writing – review and editing. **Farzad Fatehi**: conceptualization, project administration, visualization, supervision, writing – review and editing. **Sanjay Kalra**: conceptualization, project administration, visualization, supervision, writing – review and editing. **Seyed Amir Hossein Batouli**: conceptualization, project administration, visualization, supervision, writing – review and editing.

## Ethics Statement

The study was approved by the Ethics Committee of the Tehran University of Medical Sciences (Ethical Code: IR.TUMS.MEDICINE.REC.1400.1173). Written consent was obtained from all participants with potentially identifiable images or data.

## Conflicts of Interest

The authors declare no conflicts of interest.

### Peer Review

The peer review history for this article is available at https://publons.com/publon/10.1002/brb3.70484

## Data Availability

The data supporting the findings of this study are available from the corresponding author upon reasonable request.
